# Root coverage using two surgical techniques with connective tissue graft or collagen matrix: A clinical trial

**DOI:** 10.1590/0103-644020256438

**Published:** 2025-09-19

**Authors:** Luiz Eduardo Rodrigues Juliasse, Samuel Batista Borges, Karyna de Melo Menezes, Laleska Tayná Costa Barreto, Carlos Fernando Mourão, Patrícia dos Santos Calderon, Bruno Cesar de Vasconcelos Gurgel

**Affiliations:** 1 Department of Dentistry, Federal University of Rio Grande do Norte, Natal, RN, Brazil; 2 Department of Basics and Clinical Translational Sciences, Tufts University School of Dental Medicine, Boston, MA, USA

**Keywords:** gingival recession, root coverage, surgical flaps, patient morbidity, collagen matrix

## Abstract

This study compared the outcomes of Bruno (1994) and Barros et al. (2004) surgical techniques, using both subepithelial connective tissue graft (SCTG) and xenogeneic collagen matrix (CMX), for treating bilateral gingival recession type 1 defects (BR1). In this double-blind, randomized controlled trial, eighteen patients received root coverage surgeries using either Bruno (1994) or Barros et al. (2004) technique, with SCTG or CMX randomly assigned to each side. Clinical parameters, patient-reported outcomes, and post-operative pain were assessed at baseline, 3, and 6 months. Both techniques demonstrated a significant reduction in recession depth, with median root coverage exceeding 75% across all groups. The Barros technique achieved complete root coverage in 77.8% of sites for both graft types, while the Bruno technique showed complete coverage in 55.6% of CMX sites and 66.7% of SCTG sites. Significant improvements in clinical attachment level and gingival thickness were observed in all groups (p < 0.001). CMX procedures showed shorter surgical times and lower post-operative pain scores compared to SCTG. Both techniques effectively treat RT1 gingival recessions, with SCTG showing slightly superior outcomes. CMX offers advantages in surgical time and patient comfort while achieving comparable results, providing a viable alternative to the gold standard SCTG.

## Resumo

Este estudo comparou os resultados das técnicas cirúrgicas de Bruno (1994) e Barros et al. (2004) utilizando tanto enxerto de tecido conjuntivo subepitelial (ETCS) quanto matriz de colágeno xenógena (MCX) para o tratamento de defeitos bilaterais de recessão gengival tipo 1 (RT1). Neste ensaio clínico controlado randomizado duplo-cego, dezoito pacientes receberam cirurgias de recobrimento radicular utilizando a técnica de Bruno (1994) ou Barros et al. (2004), com ETCS ou MCX aleatoriamente designados para cada lado. Parâmetros clínicos, fatores centrados no paciente e dor pós-operatória foram avaliados no início do estudo, aos 3 e 6 meses. Ambas as técnicas demonstraram redução significativa na profundidade da recessão, com recobrimento radicular médio excedendo 75% em todos os grupos. A técnica de Barros alcançou recobrimento radicular completo em 77,8% dos sítios para ambos os tipos de enxerto, enquanto a técnica de Bruno mostrou recobrimento completo em 55,6% dos sítios com MCX e 66,7% dos sítios com ETCS. Melhorias significativas no nível de inserção clínica e espessura gengival foram observadas em todos os grupos (p < 0,001). Procedimentos com MCX mostraram tempos cirúrgicos mais curtos e menores escores de dor pós-operatória em comparação com ETCS. Ambas as técnicas tratam efetivamente recessões gengivais RT1 usando tanto ETCS quanto MCX, proporcionando melhorias clínicas significativas e qualidade de vida aprimorada. MCX oferece vantagens no tempo cirúrgico e conforto do paciente enquanto alcança resultados similares, embora ETCS continue demonstrando resultados levemente superiores em termos de cobertura radicular.

**Figure:**
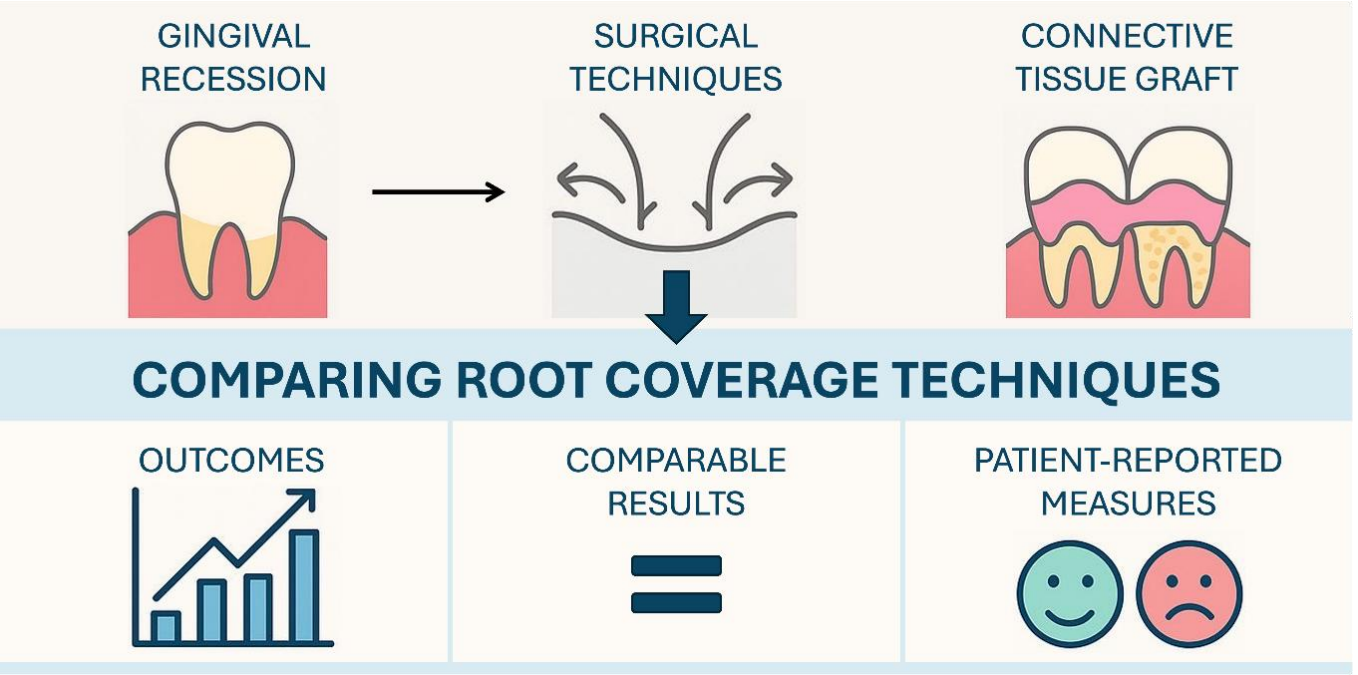

## Introduction

Gingival recession (GR) is the apical displacement migration of the marginal gingival tissues to the cement-enamel junction (CEJ) caused by various conditions, and it is associated with clinical attachment loss, potentially affecting tooth surfaces ^1^ ^,^ ^2^. This condition can result in cervical dentin hypersensitivity, root caries, an aesthetically displeasing smile appearance ^3^ ^,^ ^4^, and non-carious cervical lesions (NCCL) ^2^ ^,^ ^5^. Recent evidence suggests that GR affects almost 91.6% of the adult population on the vestibular side, decreasing to 70.7% when considering only aesthetic zones. Additionally, as GR tends to increase with age, it is estimated that 88% of the population over 65 years old and half of the population between 18 and 64 years old have at least one site with GR ^6^ ^,^ ^7^.

The treatment of exposed root surfaces has gained significance due to increasing patient aesthetic concerns and clinical implications, including dentin hypersensitivity and the risk of dental lesions ^1^ ^,^ ^5^ ^,^ ^8^. Effective coverage of exposed roots has been pursued through surgical procedures involving flaps and grafts ^9^ ^,^ ^10^, with the primary objective being to achieve stable and complete root coverage (CRC) with a tissue margin attached at the CEJ ^8^ ^,^ ^11^. These procedures also aim to significantly increase the dimensions of keratinized gingiva (thickness and width) while maintaining a healthy gingival sulcus ^1^.

The subepithelial connective tissue graft (SCTG) is considered the gold standard due to its superior root coverage percentages and increased keratinized mucosa width (KMW) ^1^ ^,^ ^5^ ^,^ ^12^. However, SCTG use presents some disadvantages, including the need for a second surgical site, limited available tissue, potential post-operative complications, and increased patient morbidity ^8^ ^,^ ^13^.To address these challenges, research has turned towards alternative materials, such as collagen matrices, aiming to reduce patient discomfort and eliminate the need for donor site harvesting ^14^ ^,^ ^15^.

The coronally advanced flap (CAF) technique is widely used in conjunction with grafts and biomaterials, including collagen matrix ^16^ ^,^ ^7^. However, adding vertical incisions to a technique has some disadvantages, such as reduced blood supply to the flap, which can interfere with graft incorporation and increase the possibility of scarring ^8^ ^,^ ^18^. Consequently, techniques that avoid such incisions, as proposed by Bruno (1994) ^10^ and Barros et al. (2004) ^19^, focused on this study, may provide better blood supply to the graft, hence accelerating the healing process and promoting root coverage, along with additional aesthetic benefits ^8^ ^,^ ^20^.

Currently, conclusive evidence indicating the superiority of one procedure over the other is lacking, underscoring the need for rigorous research to inform clinical decisions in the treatment of GR while considering patient-centered factors. Therefore, this study aims to compare the outcomes of two different techniques, Bruno (1994) and Barros et al. (2004), using both connective tissue and a xenogeneic collagen matrix for treating root coverage in single, bilateral RT1-type GR in a six-month follow-up protocol.

## Material and methods

### Study Design and Population

**Figure 1:**
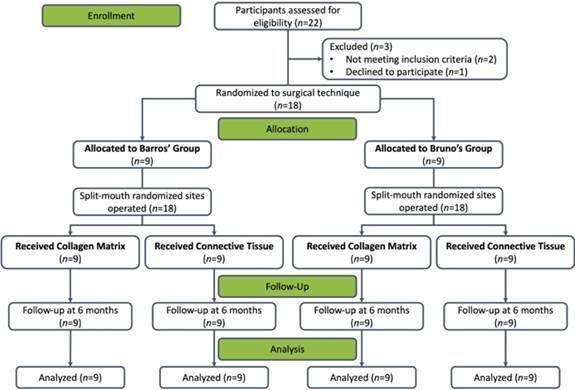
Flow chart for study selection.

This double-blind, randomized controlled trial was conducted at the Department of Dentistry, Federal University of Rio Grande do Norte (UFRN), Brazil, between February 2017 and September 2019. The UFRN Ethics Committee approved the study protocol for Human Research (approval number 1.719.095/2016), followed the CONSORT guidelines for clinical trials ^21^, and adhered to the principles of the Declaration of Helsinki (2013 revision). All participants provided written informed consent before enrollment. This investigation stems from a larger clinical trial (ClinicalTrials.gov: NCT02980055), focusing specifically on the comparison of surgical techniques and graft materials for root coverage (Figure 1).

The study population consisted of non-smoker individuals who were systematically and periodontally healthy, characterized by bleeding on probing < 10% ^1^ and probing depths ≤ 3 mm ^22^. Participants were not using orthodontic appliances or fixed or removable prostheses involving the canines and/or premolars with gingival recession. The sample was conveniently selected based on the following inclusion criteria: (a) age range between 18 and 55; (b) no prior experience with root coverage procedures; (c) diagnosis of bilateral upper RT1-type gingival recession, starting from 2 mm, according to Cairo et al. (2011) ^22^, and (d) thin gingival phenotype following the methodology proposed by De Rouck et al. (2009) ^23^. The baseline demographic and clinical characteristics of the study populations are presented in Table 1.

Randomization was performed using computer-generated random numbers in blocks of four, with allocation concealment maintained through opaque, sealed envelopes that were opened only after the patient was prepared for surgery. A total of 18 patients were randomly assigned to two groups: Bruno’s Group, which received the surgical approach proposed by Bruno (1994) ^10^, and the Barros’ Group, which received the surgical approach proposed by Barros et al. (2004) ^19^. Patients were further randomized within each group to receive either a connective tissue graft or a collagen matrix on each side of the mouth. Both procedures were performed on the same day, with the order of operations (left side *vs.* right side) randomized using a computer-generated sequence independent of the graft material allocation to minimize potential bias related to operator fatigue.

### Clinical Parameters and Outcome Measures

The clinical parameters collected included probing depth (PD), gingival recession (GR), clinical attachment level (CAL), and the width of the keratinized mucosa (KMW) of the tooth diagnosed with RT1 gingival recession ^22^. The gingival thickness (GT) and gingival phenotype (GP) were also evaluated. All parameters were assessed at baseline, 3 months, and 6 months by a blinded calibrated examiner (S.B.B.).

Examiner calibration was performed on 10 non-study patients, with measurements repeated after 48 hours, yielding high intraclass correlation coefficients for all parameters (PD: 0.655, GR: 0.722, KMW: 0.634, GT: 0.936).

**Table 1:** Baseline demographic, clinical and surgical characteristics of the study sampleAbbreviations: CMX, xenogeneic collagen matrix; SCTG, subepithelial connective tissue graft

	Barros Technique (n=9)	Bruno Technique (n=9)	p-value
Demographics			
Age (years)^*^	30.0 [26.0-33.0]	29.0 [27.0-34.0]	0.623
Gender			
Female	4	5	0.637
Male	5	4	
Recession Distribution			0.845
First premolars^†^	9 (50)	9 (50)	
Canines^†^	6 (33.3)	5 (27.8)	
Second premolars^†^	3 (16.7)	4 (22.2)	
Baseline Clinical Parameters			
*Recession depth (mm)* ^*^			
CMX sites	2.2 [2.0-2.5]	2.0 [1.8-2.3]	0.558
SCTG sites	2.3 [2.0-2.5]	2.5 [2.3-2.8]	0.445
*Keratinized tissue width (mm)* ^*^			
CMX sites	3.0 [2.0-4.0]	2.5 [2.0-3.5]	0.397
SCTG sites	3.5 [2.0-4.5]	3.0 [2.5-4.5]	0.870
Surgical Parameters			
*Surgery duration (min)* ^*^			
CMX procedures	43.0 [4.0-48.5]	38.0 [34.0-45.0]	< 0.001
SCTG procedures	75.0 [68.5-82.0]	65.0 [59.0-75.0]	0.002
Complications^‡^	1	0	-

^*^ Values presented as median [interquartile range]

^†^ Values presented as number (percentage)

^‡^ One patient experienced postoperative bleeding at the donor site

P-values were calculated using Mann-Whitney U test for continuous variables and Fisher's exact test for categorical variables

### Surgical Protocols

### Preoperative and Transoperative Procedures

Initially, all participants received oral hygiene assessments and, when necessary, underwent scaling and root planning sessions using Gracey metal curettes (Hu-Friedy, Chicago, IL, USA) to prepare for surgery. Subsequently, they received prophylaxis and hygiene instructions, emphasizing gentle brushing and care in areas with gingival recessions, and underwent periapical radiographic examinations using the parallelism technique to confirm the absence of interproximal bone loss and diagnose gingival recession type RT1, as described by Cairo et al. (2011) ^22^.

On the day of the surgical procedure, the left and right sides were randomized to receive either SCTG or CMX (Mucograft, Geistlich Pharma AG, Walhusen, Switzerland). To minimize potential bias in the patient's subjective assessment of post-operative pain, the SCTG was taken from the palate on the same side as the recipient site. The removal of the SCTG was performed using the single linear incision technique to obtain sufficient graft and minimize post-operative discomfort.

**Figure 2:**
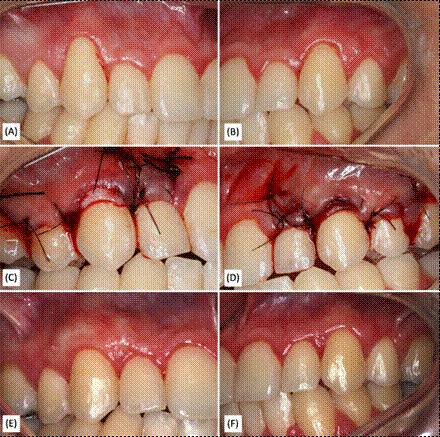
Clinical cases of gingival recessions in teeth 13 and 23, using the technique of Barros et al. (2004), on the right side (CMX) and left side (SCTG), respectively.

In the Barros group, the surgical technique of a coronally positioned extended flap was adopted (Figure 2). First, a right-angle incision was made at the base of the papilla. Then, intracellular incisions were made, followed by a deserialization of the papilla. A full-thickness flap was created, followed by a partial-thickness flap for coronal repositioning.

**Figure 3:**
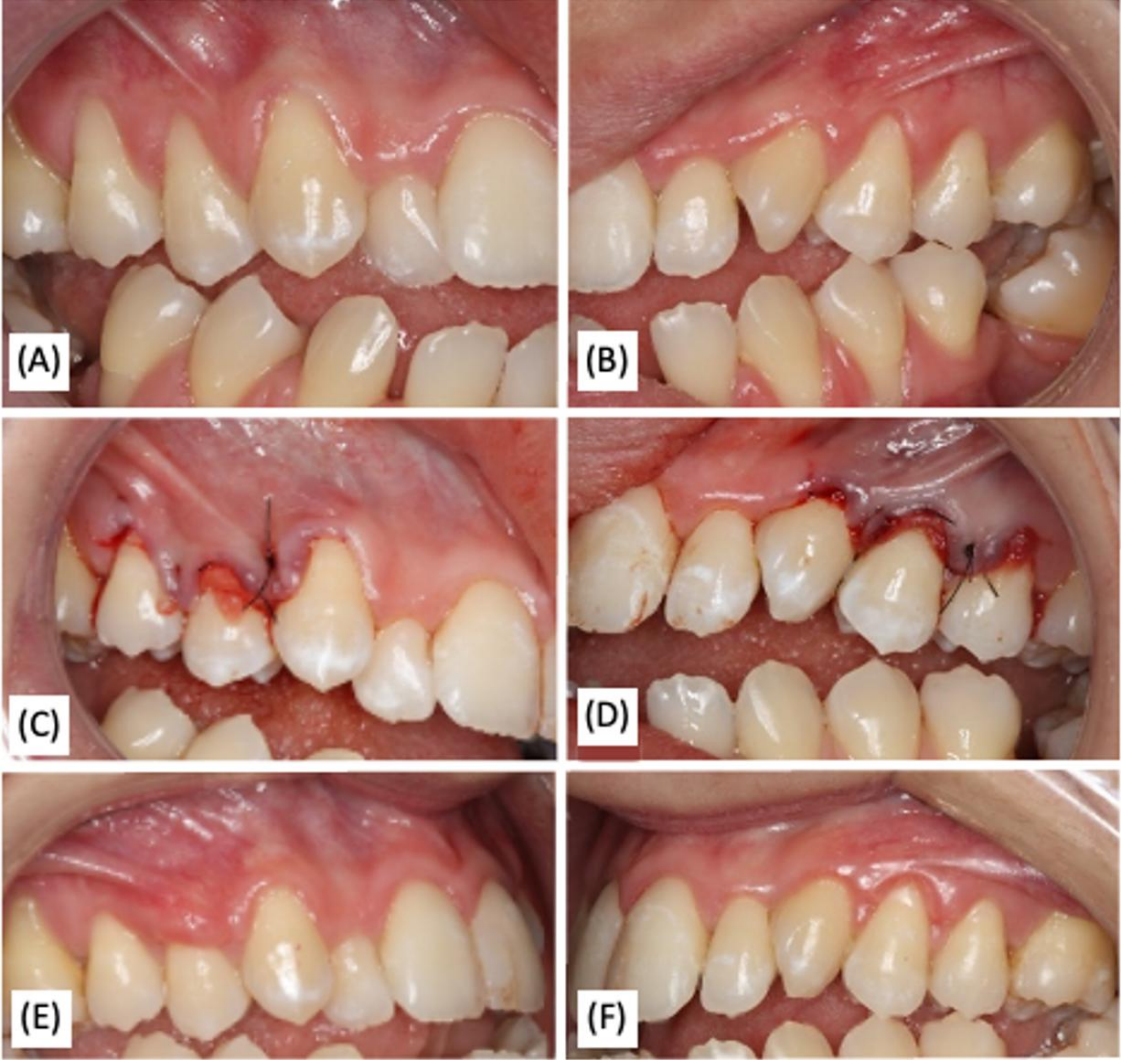
Clinical cases of gingival recessions on teeth 13 and 23, using Bruno's technique (1994), on the right (CMX) and left (SCTG) sides, respectively.

In the Bruno group, the technique involved a horizontal incision at right angles to the papilla adjacent to the recession, using a no. 15c scalpel blade (Swann-Morton, Sheffield, England, UK), at the level of the CEJ or slightly above it (Figure 3). The mesiodistal extension of the incision was increased to facilitate access to the root since no vertical incisions were made. A partial-thickness flap was created that extended apically beyond the mucogingival junction (MGJ).

### Post-operative Care

Patients were instructed to apply ice packs every 20 minutes during the first few hours after

surgery, both intra and extra orally. They were advised to avoid hot or warm foods in the first week, avoid solid foods for the first fifteen days, and abstain from brushing in the grafted area for two weeks after surgery. To clean these areas, patients were guided to rinse with 0.12% chlorhexidine gluconate mouthwash every 12 hours, 30 minutes after brushing, for 15 days.

### Patient-reported Outcomes

### Post-operative Pain Scale

**Figure 4:**
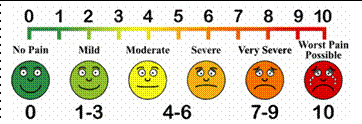
Measurement of post-operative pain using the Visual Analog Scale (VAS).

For the collection of post-operative pain data, a standardized visual analog scale (VAS) was presented to the patient (Figure 4) ^24^. Patients marked their level of post-operative pain on a scale from 0 to 10 at the three surgical sites: the two sites for the CMX and SCTG grafts and the donor site. Data were collected at 8 hours, 24 hours, 7 days, 15 days, and 30 days after the surgical procedure.

### Oral Health-Related Quality of Life (OHRQoL)

Quality of life was assessed using the OHRQoL ^25^. Through questions beginning with "What effect, if any, does the condition of your teeth, gums, and mouth have on...?" and encompassing 16 different aspects, we evaluated the quality of life-related to physical, social, and psychological dimensions. This assessment was conducted for all individuals at baseline, 3 months, and 6 months of follow-up.

### Statistical Analysis

This study was designed as a superiority trial comparing two surgical techniques and graft materials. Initial power analysis indicated that a minimum of 6 participants per group would provide 80% power to detect a difference of 9.7% in root coverage between groups, with a standard deviation of 10.2% and an alpha level of 0.05. To account for potential dropouts, the sample size was increased by 20%, resulting in 9 participants per group. The split-mouth design enhanced statistical power by reducing inter-individual variability.

Although some patients presented multiple gingival recessions, one tooth per side was selected for analysis to maintain the integrity of the split-mouth design and avoid clustering effects. When multiple eligible defects were present on one side, the tooth with the most severe recession was chosen for treatment and inclusion in the analysis. The selected tooth receiving the graft served as the unit of analysis.

Quantitative variables (PD, KMW, GT, GR, CAL, percentage of root coverage (PRC), and quality of life measures) were tested for normality using the Shapiro-Wilk test, and due to non-normal distribution, non-parametric tests were employed. Statistical significance was set at p < 0.05, with a 95% confidence level.

The primary outcome variable was the PRC, which was compared between groups (CMX *vs.* SCTG) using the Mann-Whitney U test at each time point. The Wilcoxon signed-rank test was used to assess within-group changes across follow-up times (baseline, 3 months, and 6 months), with a Bonferroni correction for multiple comparisons.

For secondary outcomes, clinical parameters (PD, GR, CAL, KMW, GT) were analyzed using the Friedman test for within-group changes over time and the Mann-Whitney U test for between-group comparisons. Post-hoc Wilcoxon signed-rank tests with Bonferroni adjustment were performed when appropriate.

Post-operative pain assessment employed the Friedman test to evaluate changes over time within each site type, while the Kruskal-Wallis test was used to compare between sites at each time point. Quality of life changes were analyzed using Friedman’s test for repeated measures, with Bonferroni correction for multiple comparisons. Categorical variables were analyzed using Chi-square tests or Fisher’s exact test when expected frequencies were less than 5.

Results are presented as median [interquartile range] for continuous variables and frequencies (percentages) for categorical variables. All statistical analyses were performed using SPSS version 23.0 (Chicago, IL, USA), with graphics generated using PyCharm 2024.1.1.

## Results

### Study Population and Protocol Adherence

All eighteen enrolled participants (9 females, nine males; median age 29.0 [27.0-33.5] years) completed the study protocol without losses to follow-up (Figure 1). While some patients presented multiple gingival recessions, one tooth per side was selected for analysis following pre-established criteria. The distribution of treated teeth and baseline clinical parameters were similar between groups (Table 1). Only one adverse event occurred throughout the study: post-operative bleeding at a donor site in the Barros technique group, which resolved without complications.

### Surgical Procedures and Clinical Outcomes

The surgical time was significantly shorter for CMX procedures compared to SCTG in both techniques (p < 0.001) (Table 2). At 6 months post-surgery, both techniques significantly reduced recession depth from baseline (p < 0.001). The Barros technique achieved complete root coverage in 77.8% of sites for both graft types, while the Bruno technique showed complete coverage in 55.6% of CMX sites and 66.7% of SCTG sites.

When comparing materials regardless of technique, SCTG demonstrated higher median root coverage (85.5% [78.0-93.0%]) compared to CMX (76.7% [69.0-85.0%]; p = 0.042). Within each technique, SCTG also performed better than CMX (Barros technique: 86.9% [79.0-94.0%] *vs.* 77.2% [70.0-86.0%], p = 0.048; Bruno technique: 84.0% [76.0-92.0%] *vs.* 76.2% [68.0-84.0%], p = 0.046).

### Soft Tissue Parameters

Both techniques significantly improved gingival thickness (p < 0.001). The proportion of sites converting from thin to thick phenotype was similar between techniques with SCTG (Barros: 55.6%, Bruno: 66.7%), while CMX showed slightly lower conversion rates (Barros: 55.6%, Bruno: 44.4%). Keratinized tissue width increased significantly in the Barros group for both CMX (p = 0.014) and SCTG (p < 0.001), while changes in the Bruno group were not statistically significant (p = 0.807) (Table 2).

### Clinical Attachment Level Changes

Clinical attachment level (CAL) improved significantly across all treatment groups over the 6-month follow-up period. At baseline, median CAL values were 2.8 mm [2.0-4.0] for the Bruno/CMX group, 3.2 mm [2.5-4.5] for Bruno/SCTG, 3.5 mm [2.8-4.8] for Barros/CMX, and 3.9 mm [3.0-5.0] for Barros/SCTG. By 6 months, each group demonstrated a statistically significant reduction in CAL (p < 0.001), with median values decreasing to 1.5 mm [1.0-2.0], 1.7 mm [1.2-2.3], 1.8 mm [1.3-2.4], and 1.9 mm [1.0-2.5], respectively. Despite these within-group improvements, no statistically significant differences were observed between surgical techniques (p = 0.689) or graft materials (p = 0.722) (Table 2).

### Patient-Centered Outcomes

**Figure 5:**
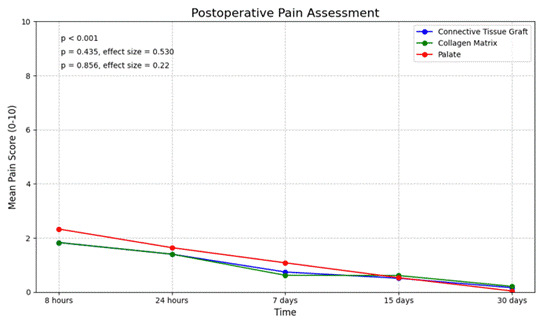
Postoperative pain assessment. Postoperative pain scores for Connective Tissue Graft, Collagen Matrix, and Palate (donor site) at 8 hours, 24 hours, 7 days, 15 days, and 30 days post-surgery. Significant differences between techniques were observed (p < 0.001), with Connective Tissue Graft showing the highest initial pain scores, followed by Collagen Matrix and Palate. Pain scores gradually decreased over time, reaching low levels by day 30 for all techniques.

Post-operative pain decreased significantly over time at all surgical sites (p < 0.001). Pain scores were initially higher at donor sites (median 2.0 [0.0-4.0]) compared to recipient sites (CMX: 1.0 [0.0-3.0]; SCTG: 1.0 [0.0-3.0]) at 8 hours post-surgery but decreased to minimal levels (VAS < 1) by day 15 across all sites. When comparing donor sites with CMX sites at each time point, donor sites showed significantly higher pain scores than CMX sites at 8 hours, 24 hours, and 7 days (p < 0.05). CMX procedures consistently showed lower pain scores compared to SCTG, though this difference was not statistically significant (p = 0.367) (Table 3) (Figure 5).

While absolute pain scores were generally low (VAS < 2), especially after 15 days, the statistically significant differences reflect consistent patterns across participants rather than clinically meaningful differences at later time points. These findings should be interpreted in the context of overall low post-operative morbidity for all procedures.

**Figure 6:**
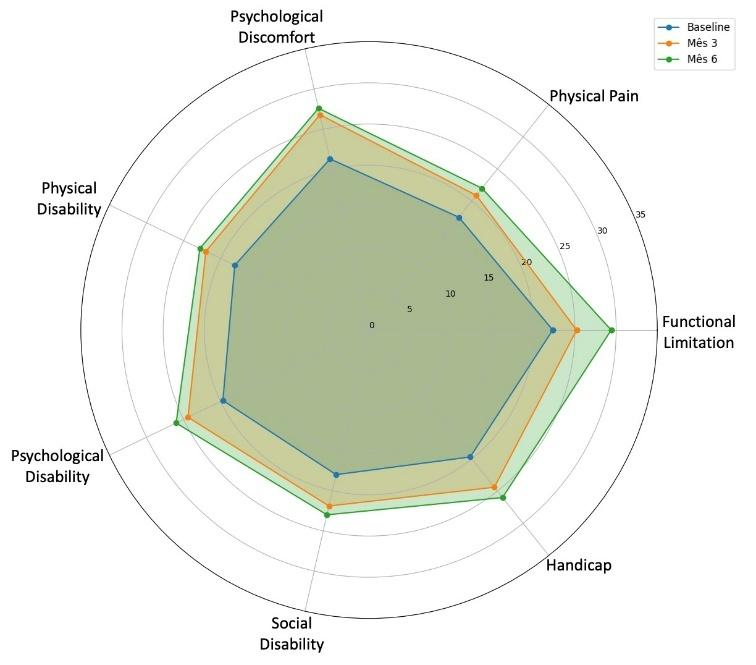
OHIP-14 scores across seven domains at baseline, 3 months, and 6 months post-surgery, demonstrating improvement in oral health-related quality of life over time. The radar chart illustrates the increasing sum of means and medians for each domain, represented by data points moving outward from the center at each time point. This pattern indicates a notable and proportional enhancement in patients' quality of life for both techniques. The improvement in OHIP-14 scores was statistically significant from baseline to 3 and 6 months (p < 0.001) for all domains, with no significant differences between the 3- and 6-month time points or between the two surgical techniques.

Quality of life improved significantly across all domains from baseline to 6 months (p < 0.001). The physical domain showed the most pronounced improvement, particularly in the Barros technique group (baseline: 22.0 [20.0-24.0]; 6 months: 29.0 [28.0-30.0]). Both techniques demonstrated comparable improvements in social and psychological domains by the 6-month follow-up (p = 0.852) (Table 4) (Figure 6).

### Comparative Effectiveness

Overall, both surgical techniques demonstrated clinical effectiveness with either graft material. The Barros technique showed slightly higher percentages of complete root coverage and greater gains in keratinized tissue width, while the Bruno technique offered the advantage of shorter surgical times (Table 2). SCTG procedures resulted in better root coverage percentages compared to CMX, confirming its status as the gold standard. However, CMX procedures were associated with shorter surgical times and lower post-operative pain scores, though these differences did not reach statistical significance (Tables 2and3) (Figure 5).

**Table 2:** Clinical outcomes at 6 months after root coverage procedures comparing Bruno and Barros techniques with either xenogeneic collagen matrix (CMX) or subepithelial connective tissue graft (SCTG) in a split-mouth design.

	Barros Technique		Bruno Technique		p-value^*^
	CMX	SCTG	CMX	SCTG	
Root Coverage					
Complete coverage^†^	7 (77.8%)	7 (77.8%)	5 (55.6%)	6 (66.7%)	0.215
Mean coverage^‡^	77.2% [70.0-86.0%]	86.9% [79.0-94.0%]	76.2% [68.0-84.0%]	84.0% [76.0-92.0%]	0.558
Material comparison p-value^§^	0.048		0.046		
Residual recession^‡^	0.5 mm [0.0-1.0 mm]	0.3 mm [0.0-0.5 mm]	0.5 mm [0.0-1.0 mm]	0.4 mm [0.0-0.8 mm]	0.689
Tissue Changes					
Δ Keratinized width^‡^	1.5 mm [1.0-2.0 mm]	1.8 mm [1.0-2.5 mm]	0.8 mm [0.5-1.0 mm]	0.6 mm [0.0-1.0 mm]	0.348
Within-group p-value^¶^	0.014	< 0.001	0.096	0.107	
Δ Gingival thickness^‡^	0.8 mm [0.5-1.0 mm]	0.9 mm [0.7-1.2 mm]	0.5 mm [0.3-0.8 mm]	1.0 mm [0.7-1.3 mm]	0.83
Within-group p-value^¶^	< 0.001	< 0.001	< 0.001	< 0.001	
Phenotype conversion to thick^†^	5 (55.6%)	5 (55.6%)	4 (44.4%)	6 (66.7%)	0.003

Abbreviations: CAL, clinical attachment level; CMX, xenogeneic collagen matrix; KMW, keratinized mucosa width; SCTG, subepithelial connective tissue graft

^*^ p-value for comparison between techniques (Mann-Whitney U test)

^†^ Values presented as number (percentage) (Chi-square test)

^‡^ Values presented as median [interquartile range]

^§^ p-value for comparison between materials within technique (Mann-Whitney U test)

^¶^ p-value for change from baseline to 6 months (Wilcoxon signed-rank test)

Δ represents change from baseline to 6 months

**Table 3:** Patient-reported pain outcomes at different time points after root coverage procedures using visual analog scale (VAS, 0-10) across treatment sites.

Time Point	Pain Scores (VAS 0-10)^*^			p-value^†^	p-value^‡^
	CMX sites	SCTG sites	Donor sites		
8 hours	1.0 [0.0-3.0]	1.0 [0.0-3.0]	2.0 [0.0-4.0]	< 0.001	0.024
24 hours	1.0 [0.0-2.0]	1.0 [0.0-2.5]	1.5 [0.0-3.0]	< 0.001	0.038
7 days	0.0 [0.0-1.0]	0.5 [0.0-1.0]	1.0 [0.0-2.0]	< 0.001	0.043
15 days	0.0 [0.0-1.0]	0.0 [0.0-1.0]	0.0 [0.0-1.0]	< 0.001	0.642
30 days	0.0 [0.0-0.0]	0.0 [0.0-0.0]	0.0 [0.0-0.0]	< 0.001	0.891

ABBREVIATIONS: CMX, XENOGENEIC COLLAGEN MATRIX; SCTG, SUBEPITHELIAL CONNECTIVE TISSUE GRAFT; VAS, VISUAL ANALOG SCALE (0 = NO PAIN, 10 = EXTREME PAIN)

^*^ Values presented as median [interquartile range]

^†^ p-value for change over time (Friedman test)

^‡^ p-value for comparison between sites at each time point (Kruskal-Wallis test)

^§^ p-value for comparison between CMX and SCTG across all time points (Mann-Whitney U test)

**Table 4:** Oral Health-Related Quality of Life scores across physical, social, and psychological domains at baseline, 3 months, and 6 months after root coverage procedures.

Domain	Baseline	3 Months	6 Months	p-value^*^
Physical				< 0.001
Barros technique	22.0 [20.0-24.0]	25.0 [24.0-27.0]	29.0 [28.0-30.0]	
Bruno technique	21.0 [19.0-23.0]	27.0 [25.0-28.0]	28.0 [26.0-29.0]	
Technique comparison p-value^†^	0.425	0.368	0.382	
Social				< 0.001
Barros technique	17.5 [16.0-19.0]	21.0 [20.0-22.0]	22.0 [21.0-23.0]	
Bruno technique	18.5 [17.0-20.0]	22.0 [21.0-23.0]	23.0 [22.0-24.0]	
Technique comparison p-value^†^	0.473	0.298	0.352	
Psychological				0.003
Barros technique	21.0 [19.0-23.0]	27.0 [25.0-28.0]	28.0 [26.0-29.0]	
Bruno technique	18.0 [16.0-20.0]	22.0 [20.0-24.0]	23.0 [21.0-24.0]	
Technique comparison p-value^†^	0.085	0.062	0.072	

Values presented as median [interquartile range]. Higher scores indicate better quality of life. Physical domain scores range from 7-35, social domain from 5-25, and psychological domain from 5-25, with all domains showing significant improvement from baseline to 6 months

^*^ p-value for change over time (Friedman test)

^†^ p-value for comparison between techniques at each time point (Mann-Whitney U test)

## Discussion

This study evaluated the clinical outcomes and patient-centered results of two surgical techniques (Bruno and Barros) using either SCTG or CMX for root coverage of RT1 gingival recessions. Our findings demonstrate that both techniques can achieve significant improvements in clinical parameters, with SCTG consistently providing slightly superior results in terms of root coverage, confirming its position as the gold standard. However, CMX represents a viable alternative that reduces surgical time and patient discomfort while providing acceptable clinical outcomes.

The complete root coverage rates observed in our study (55.6-77.8%) align with previously reported outcomes in the literature. The slightly higher complete root coverage achieved with the Barros technique might be attributed to its enhanced flap design, though this difference did not reach statistical significance. Our results are consistent with recent systematic reviews that report success rates for various root coverage procedures ^5^ ^,^ ^16^ ^,^ ^17^.

A key finding of our study was the significant improvement in gingival thickness across all groups, with successful conversion from thin to thick phenotype in more than half of the treated sites. This phenotype modification holds particular relevance as recent evidence indicates its dual role in preventing recession recurrence and protecting against the development of non-carious cervical lesions on exposed root surfaces ^14^ ^,^ ^24^. The comparable thickness gains achieved with CMX represent a significant advantage, considering that obtaining sufficient SCTG material can be challenging in patients with thin palatal mucosa.

The long-term stability of root coverage procedures remains a fundamental consideration in periodontal plastic surgery. According to several studies, gingival recession defects tend to recur over time when using a flap procedure alone compared to bilaminar procedures ^5^ ^,^ ^12^. There is a direct relationship between the amount of keratinized mucosa width, gingival thickness, and treatment stability at 6-12 months post-surgery. Our results showed significant improvements in these parameters across all groups, suggesting potential long-term stability; however, a longer follow-up would be valuable to confirm this hypothesis.

Our patient-reported outcomes revealed advantages of CMX procedures, including consistently lower post-operative pain scores, faster recovery times, and elimination of donor site morbidity. These findings support previous research ^1^ ^,^ ^5^ ^,^ ^8^ and suggest specific benefits for patients with thin palatal mucosa ^13^ since surgeons can apply thicker grafts using CMX ^15^. The significant reduction in surgical time (p < 0.001) further strengthens the clinical applicability of CMX procedures.

Both techniques demonstrated unique advantages in handling different graft materials. The success of the Barros technique appears to be linked to the strategic placement of relaxing incisions ^19^, whereas the effectiveness of the Bruno technique relates to precise flap positioning ^10^. These technical nuances, critical when using CMX, reflect an evolution from the original protocols developed for autogenous grafts.

While our sample size provided adequate power for detecting clinically meaningful differences, larger studies might reveal subtle variations between techniques. The split-mouth design enhanced internal validity by controlling patient-related variables. Although the 6-month follow-up is comparable to similar studies, it represents an intermediate-term evaluation; longer-term data would be valuable for assessing stability.

The comparable outcomes between techniques and materials have several implications for clinical practice. Both surgical approaches can be confidently employed for RT1 recessions, with CMX providing a viable alternative to SCTG, especially advantageous for patients with limited palatal tissue. Technique selection can be based on individual case characteristics and operator preference.

This clinical trial concludes that both the Bruno and Barros surgical techniques are effective in treating gingival recessions using either SCTG or CMX, with no statistically significant differences between them. Although designed as a superiority trial, the findings suggest therapeutic equivalence between techniques. CMX, in combination with either approach, represents a practical alternative for managing single gingival recessions. Nonetheless, SCTG continues to show slightly superior results in terms of root coverage, confirming its status as the gold standard. These conclusions are supported by clinical improvements observed over the six-month follow-up period, including reduced gingival recession, increased clinical attachment levels, shorter surgical times, and enhanced patient quality of life.
